# Simulating ozone degradation of deoxynivalenol and its bio-safety assessment by mouse model

**DOI:** 10.3389/fmicb.2023.1286503

**Published:** 2023-10-30

**Authors:** Chao Sun, Fang Yang, Jianhui Xiao, Wenwen Zhou, Jun Li, Xiaolong Gu

**Affiliations:** ^1^College of Food Science and Engineering, Jiangxi Agricultural University, Nanchang, China; ^2^College of Veterinary Medicine, Yunnan Agricultural University, Kunming, China; ^3^Technology Center of Nanchang Customs District, Nanchang, China

**Keywords:** deoxynivalenol, simulating ozone degradation, safety assessment, *in vivo* toxicity, mouse model

## Abstract

Deoxynivalenol (DON), a trichothecene mycotoxin, is one of the most prevalent mycotoxins globally, primarily produced by *Fusarium* species. DON exposure could cause a range of symptoms, including nausea, vomiting, gastroenteritis, growth retardation, immunosuppression, and intestinal flora disorders in both humans and animals. Recently, ozone degradation technology has been applied for DON control. However, the safety of the contaminated grain after degradation was often ignored. Therefore, the implementation technology for assessing the safety of DON-contaminated grain degradation is of great significance for food safety. In this study, based on previous degradation result of DON, we further studied and assessed the toxicity of corn contaminated with ozone-degrading DON by animal experiments in mice. We simulated feed made from corn contaminated with DON produced by inoculated *Fusarium graminearum*, which was treated with an ozone aqueous solution. DON treated by ozone could effectively increase the expression of total protein in mice and improve the immune system efficacy. Meanwhile, compared with DON directly-exposed mice, the corn with degrading DON could effectively maintain the level of liver and kidney immune function, and improved growth performance, enterohepatic circulation, and energy metabolism. Our study indicated that the toxicity of fed corn contaminated with degrading-DON decreased significantly after ozone degradation, resulting in a much lower toxicity compared to the DON group, or nontoxicity to some extent. Therefore, we hope that this mouse model could be used as a promising approach for assessing the risk of fungal toxins on metabolism, immunity, and intestinal health.

## 1. Introduction

Deoxynivalenol (DON), a typical type B trichothecene toxin, which is a secondary metabolite produced by *Fusarium graminearum* (Pestka and Smolinski, 2005). It’s one of the most commonly-encountered toxins in cereal crops, in terms of both frequency and concentration in corn, wheat, oats, and barley throughout the world (Sun et al., 2017). DON can accumulate in cereals, as a mycotoxin that remains stable at 120°C and partially stable at 210°C, allowing it to persist for years in different environments (Khaneghah et al., 2018). When present in food, DON can cause a range of symptoms in consumers and livestock, including vomiting, gastroenteritis, and immunosuppressive effects (Chen et al., 2018; Sun et al., 2020). At the cellular and molecular level, DON binds to ribosomes, inhibits the synthesis of protein, RNA, and DNA, and also induces cell apoptosis (Zhao et al., 2022; Zha et al., 2023). In addition to direct acute toxicity, DON toxins also exhibit cellular and immune toxicity (Deng Y. et al., 2023). Eating food contaminated with DON can easily cause serious damage to human and animal health, especially to organs such as the liver, kidneys, and intestines (Hou et al., 2023). Through medical microscopy, it can be observed that liver cell particles may exhibit degeneration symptoms. In severe cases, liver tumors and other forms of liver damage may also occur (Awad et al., 2019; Deng H. et al., 2023). Additionally, the ingestion of deoxynivalenol over a long period, even at low concentrations, has been shown to adversely affect the health of both humans and animals (Graziani et al., 2015). The production of DON toxins is actually closely related to seasonality and regionality (Edwards, 2009). Due to the influence of warm and humid climate in certain regions, such as the Yangtze River Basin, cereal DON pollution is severe. This ultimately leads to a higher incidence of certain cancer diseases in the local area compared to other regions, such as liver cancer and intestinal cancer (Al-Jaal et al., 2019). Therefore, it is of great significance to conduct safety evaluation research on food contaminated by DON.

In our previous studies, aqueous ozone (ozone at 80 mg L^−1^) was used to detoxify a DON standard (15 mg L^−1^) for 7 min, and the degradation rate reached 83% (Sun et al., 2017). This method was one of the useful degradation driven by ozone for the detoxification of DON in aqueous systems.

In order to further analyze the safety of DON degradation, this paper simulates the process of *F. graminearum* infecting corn, conducts animal feeding experiments, and investigates its toxicity to ensure safety. The *in vivo* safety evaluation experiment shall be conducted in accordance with the 28 days oral toxicity test method recommended by the China National Standard GB 15193.22-2014. We inoculated *F. graminearum* F7875 producing the toxin onto corn niblet for a simulated infection experiment. Then corn niblets contaminated with DON, before and after treatment with ozone water. The treated niblets was then used to create a mouse diet feed based on a specific formula and administered to the mice. The experiment was conducted continuously for 28 days. Then, the toxicity of ozone-degrading DON was analyzed and evaluated by studying the changes in the health of Kunming mice. This analysis was conducted by examining blood biochemical indicators and organ tissue pathological changes before and after exposure. The above methods could effectively evaluate the safety of contaminated food toxicity after DON detoxification.

The main purpose of this study is to: (i) simulate the process of *F. graminearum* infecting corn niblet; (ii) develop feed formulas and feeding methods of detoxified DON by contaminating corn; and (iii) evaluate the *in vivo* safety of DON contaminated corn by using a mouse model.

## 2. Materials and methods

### 2.1. Materials and reagents

DON standards were purchased from Sigma-Aldrich (St. Louis, MO, United States), CAS: 51481-10-8; acetonitrile and methanol (chromatographic grade) were purchased from by Sigma and Fisher Scientific (CA, United States). Stock solutions of the standard analyte were prepared in methanol at concentrations ranging from 1–20 mg L^−1^. Diluted standard working solutions (1–10 mg L^−1^) were stored in darkness at 4–6°C. For the UPLC-Q-TOF studies, ultra-pure water (resistivity ≥18 MΩcm^−1^) was obtained from a Millipore-Q SP Reagent Water system (Millipore, Bedford, MA, United States) and filterated through a 0.22 μm filter. Other chemicals were of analytical grade.

*F. graminearum* F7875 (CFCC 7875: Gibberella zeae (Schwein.) Petch) was purchased from the National Strain Collection Center; F7875 medium: using solid medium – potato glucose agar medium (PDA).

### 2.2. Extraction and cleanup of DON

Extraction of DON from corn samples and purification with the multifunctional purification column were carried out according to the modified procedure of China National Standard (GB/T 23503-2009). Twenty-five grams of milled samples were placed in a 250 mL vial, and 100 mL of acetonitrile water (80:20 v/v) was added to the vial. The vial was then shaken on the oscillator at 150 rpm for 30 min, and the supernatant was separated under vacuum through filter paper. An aliquot of 5 mL of the extract was passed through a multifunctional purification column (DonStar R 82O-COIAC5004 United States), and 3 mL of the purified extract was collected. The eluent was transferred to a vial, mixed, and dried under nitrogen gas streams at 65°C. The residue was then re-dissolved with a 200 mL water mixture containing methanol (80:20, v/v). And the vial was shaken on the vortex mixer for 15 s. Thus, the solution was centrifuged at 10,000 rpm for 5 min. Finally, the supernatant was collected into a vial and stored at 4°C until UPLC-TQD-MS analysis.

### 2.3. Infection of *F. graminearum* on maize

*F. graminearum* F7875 was inoculated onto corn niblet for solid cultures to observe the infection status of *F. graminearum*. The corn niblets were inoculated by *F. graminearum* after UV irradiation, then mixed it and cultured in a 32°C incubator in dark environment. The inoculated niblets were shaken 10 times every 3 days during the 30 days incubation to ensure *F. graminearum* growth. During 30 days of cultivation, we continuously observe the infection of *F. graminearum* on corn niblet.

### 2.4. Animal experiments

According to the 28 days oral toxicity test standard of GB 15193.22-2014, 40 healthy Kunming mice (half male and half female, 5 weeks aged) of clean quality were selected for the experiment. The male mice weighed 33 ± 2 g, while the female mice weighed 28 ± 1 g. They were purchased from the Suzhou Experimental Animal Mouse Center.

The mice were raised in a humane manner. All experimental operations followed the ethical guidelines of the European Community (2010/63/EU). Animal handling and experimental protocols were conducted strictly according to the guidelines of the institutional animal ethics committee. The feed of mice satisfied relevant hygiene standards for animal feed used in experimental in the experiment.

### 2.5. Configuration of experimental feed

The customized feed formula of mouse is processed by Jiangsu Collaborative Pharmaceutical Bioengineering Co., Ltd. The feed formula and grouping situation of mice are shown in Tables 1, 2. The corn was purchased from supermarkets. The first group served as the control group, for which we directly prepared corn kernels as feed without any treatment; the second group was treated by the niblets with ozone water at a concentration of 80 mg/L for 10 min, and then we used these processed niblets to prepare feed; in the third group, the niblets were inoculated with *F. graminearum* and cultured for 30 days to form DON-contaminated corn. The DON-contaminated corn was used to produce feed (DON at a concentration of 1.5–2 mg/L); the fourth group was fed by the DON-contaminated corn with ozone water at a concentration of 80 mg/L for 10 min. The study adhered to the appropriate hygiene standards for the animal feed utilized in the experiments.

**Table 1:** Customized feed of mice.

Raw material	Proportion (%)
corn niblet	52.92
soybean meal	25.0
wheat flour	9.0
wheat bran	7.0
sodium chloride	0.2
calcium hydrogen phosphate	1.15
calcium carbonate	1.65
calcium sulfate	1.18
zinc sulfate	1.2
ferrous sulfate	0.5
multivitamin	0.2

**Table 2:** Group information of animal experiment.

Description	Group
Corn niblets (control)	group 1
Ozone treatment of niblets	group 2
DON-contaminated niblets	group3
Ozone-degrading DON-contaminated niblets	group 4

### 2.6. Animal feeding

Mice were fed with a basal diet for 1 weeks in the animal experimental room. This was done to isolate and culture them to acclimate them to the environment. The feeding temperature and the humidity of mouse were 22 ± 2°C and 50 ± 10%, and the periodic illumination and darkness were both 12 h. In the mouse breeding room, mice were randomly divided into 8 cages and 4 groups. The group situation (2 cages in each group and 5 male/female mice in each cage) is shown in Table 2; Ji et al. (2017). Water was freely available. Mice were fed by a twice-daily diet of 4 ± 1 g.

### 2.7. Collection of blood and tissue samples

Mice were fasted for 12 h before blood collection and slaughter. Blood was collected from the eyeball and the mice were euthanized by cervical dislocation (Yang et al., 2019). The blood of mice was collected and we allowed it to settle in a sterile centrifuge tube for 2 h. It was then centrifuged at 3500 rpm for 10 min. We acquired the serum and stored it at −80°C for further testing (Seyed Toutounchi et al., 2021). For collection of tissue samples, the mice were dissected and the necessary internal organs were extracted for testing purposes. Subsequently, the total weight of the mice extracted organs was measured. The specimens of mice were rinsed with physiological saline and preserved in a formaldehyde solution for tissue slicing (Ye et al., 2023). The biochemical indicators for detecting serum and liver function in mice include: Alanine transaminase (ALT), Aspartate aminotransferase (AST), and Blood urea nitrogen (BUN) (Bai et al., 2021).

### 2.8. Weight measurement of mice and histopathology

Firstly, mice were weighed one by one and the initial weight was recorded. During the experiment, we weighed them every 10 days to record weight changes, and their feed consumption were also recorded (Yang et al., 2019).

The tissue was embedded in paraffin, cut into 5 μm sections using a Leica Pathology Microtome, and stained with hematoxylin-eosin. Representative micrographs of the jejunum sections were obtained using a 20× objective with an inverted fluorescence microscope. The hematoxylin-eosin staining (HE) staining and slicing methods of various tissues and organs in mice refer to the methods used by Zhou et al. (2021).

## 3. Results

### 3.1. Infection of *F. graminearum* in maize

**Figure 1:**
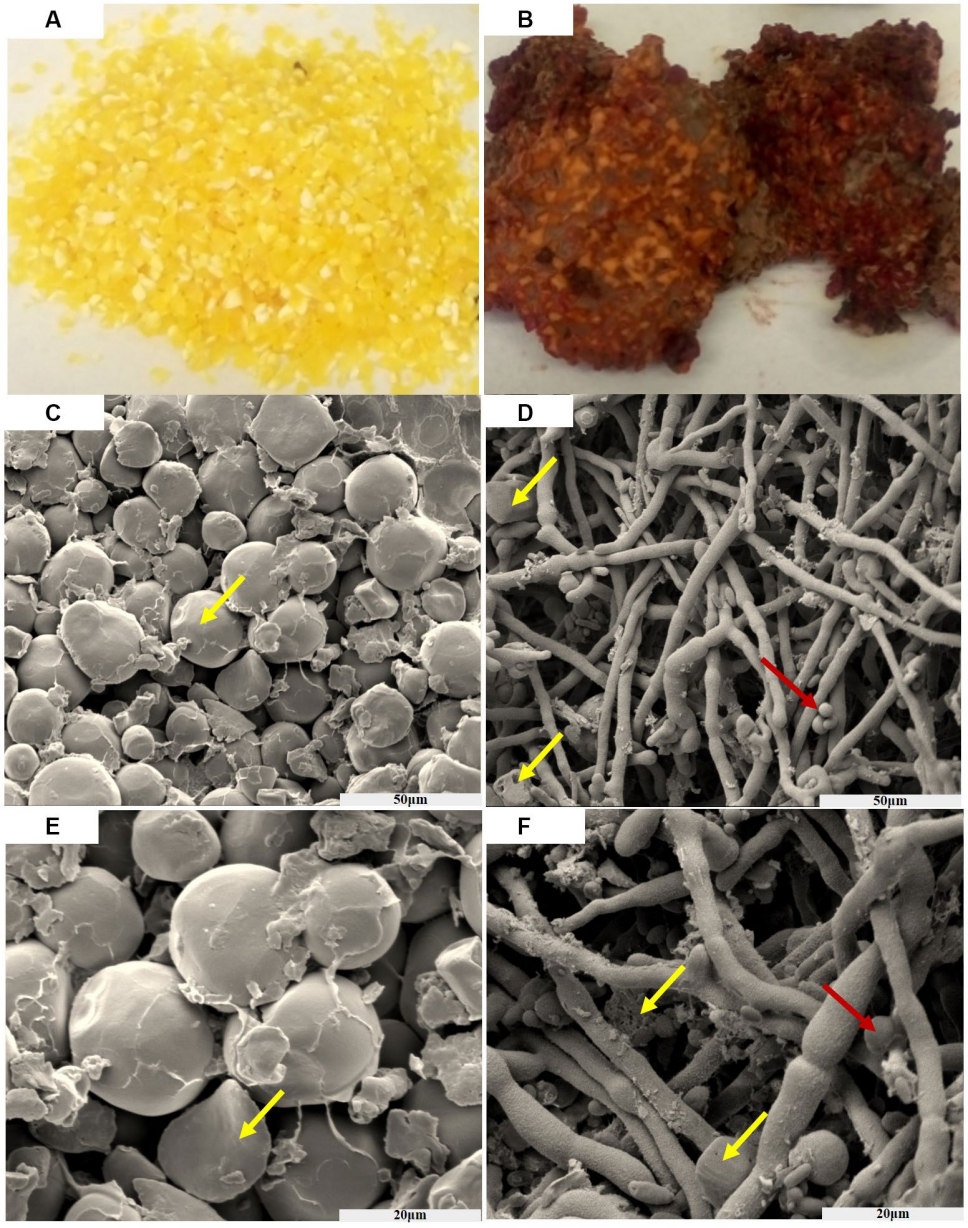
The process of corn infected by *F. graminearum*, before infection **(A)**, after infection **(B)**. Scanning electron microscopy of the corn before AND after infection (50 μm) **(C,D)**. Scanning electron microscopy of the corn before AND after infection (20 μm) **(E,F)**. Yellow arrows indicate corn starch granules, and red indicates spores of *F. graminearum*.

From Figure 1, it can be observed that the texture of the golden corn particles undergo significant changes after being infected by *F. graminearum*. After suffering infection, the previously intact and granular corn has transformed into fragmented powder, and bound together by mycelium. Furthermore, the previously golden coloration has undergone a transition, resulting in a brown or reddish hue.

After subjecting the corn to a constant temperature of 32°C and keeping it in the dark for 30 days, a noticeable color change from yellow to brick red-brown was observed. Additionally, the concentration of deoxynivalenol (DON) was measured to be within the range of 2–4 mg/kg. The entire fragmented powder is interconnected in a way that resembles the dough, and it emits a musty odor smelling sawdust. As time progressed, the concentration of toxins increased.

Figure 1 show scanning electron micrographs of *Fusarium* infected corn niblet, while Figures 1C,E show the state of corn niblets at different scales before infection. Through photographing, it can be observed that niblets are oval shaped starch particles before infection, with a smooth surface and a rounded luster. The particles are closely arranged and there are certain gaps between them. Figures 1D,F show the infection status of *F. graminearum* at different scales. After being completely infected, significant changes were observed in the morphology of corn niblet. The surface of starch granules was completely enveloped by tight mycelium, and the previously round starch granules disappeared. Most of the granules were broken down into fragments and smaller granules, while the mycelium exhibited coarser morphological characteristics. After being consumed by the mycelium, starch particles highlight a porous structure, and the decomposed starch particles adhere to the surface of the mycelium. *F. graminearum* has already undergone growth and maturation, predominantly displaying spore structures. These phenomena indicates that *F. graminearum* presents high toxic, and it is difficult for DON-contaminated corn niblets to retain any edible value after being complete infection.

### 3.2. The effect of each experimental group on the body weight of mice

All mice survived except for one mouse from group 3 during the study. The bodyweight parameters of the four groups of mouse were compared over a 4 weeks experimental period shown in Tables 3 4. The body weight results of male and female mice in each group after a 0–28 days’ feed are shown in Tables 3 4. It can be seen that after feeding with different groups, there is no significant difference in body weight between the first group of corn raw materials and the second group of ozone treated corn. However, the body weight of mice in the third group of toxin feed is lightest among these four groups, and its variants is lower than that of the previous two groups. This finding suggests that the toxin remained active during the feeding process in the group inoculated with DON, resulting in a significantly detrimental effect on their health.

**Table 3:** Weight changes of male mice in experimental groups.

Weight (piece/day)	Group 1 (g)	Group 2 (g)	Group 3 (g)	Group 4 (g)
0	32.2 ± 1.1	33.8 ± 1.3	33.2 ± 1.2	33.4 ± 1.9
10	35.3 ± 1.0	36.7 ± 1.2	35.4 ± 1.1	36.2 ± 1.4
20	39.6 ± 1.3^a^	39.7 ± 1.0^a^	36.3 ± 1.5^b^	38.2 ± 1.3^ab^
30	42.5 ± 2.6^a^	43.2 ± 1.4^a^	37.8 ± 1.6^c^	40.5 ± 1.2^b^

Groups with differing letters are significantly different (adjusted *p* < 0.05).

**Table 4:** Weight changes of female mice in experimental groups.

Weight (piece/day)	Group 1 (g)	Group 2 (g)	Group 3 (g)	Group 4 (g)
0	30.6 ± 1.2	29.6 ± 1.3	29.2 ± 1.2	29.4 ± 1.9
10	32.3 ± 1.9	33.7 ± 1.7	31.4 ± 1.1	32.3 ± 1.4
20	35.8 ± 1.3^a^	35.7 ± 1.2^a^	33.3 ± 1.8^b^	34.2 ± 1.3^ab^
30	38.6 ± 1.4^a^	38.2 ± 1.4^a^	34.5 ± 1.6^b^	35.2 ± 1.6^b^

Groups with differing letters are significantly different (adjusted *p* < 0.05).

**Figure 2:**
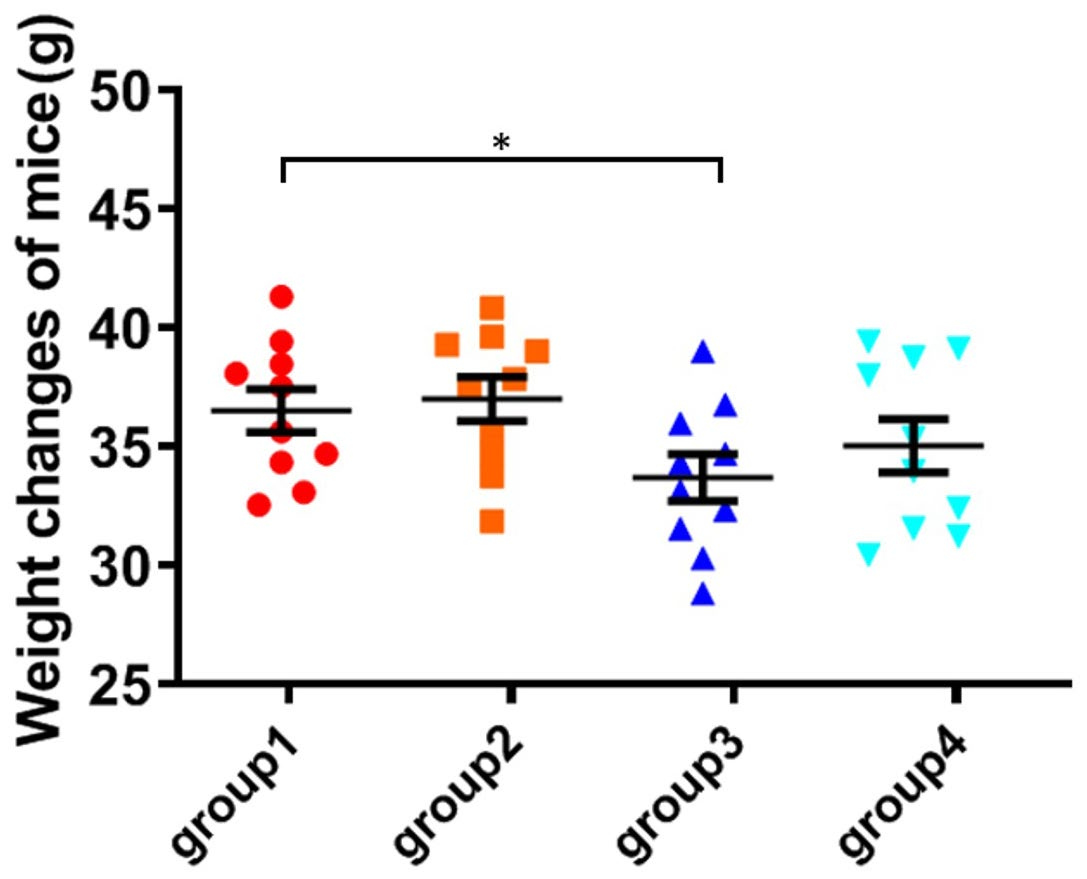
Weight changes of mice. The data is expressed as mean ± standard deviation. *p < 0.05 is significant, analyzed by one-way ANOVA test.

The weight of the fourth group of ozone-treatment was higher than that of the third group. This observation suggests a significant reduction in the toxicity of DON after ozone degradation, with little impact on the mice weight (Figure 2).

### 3.3. The effect of DON toxin on the liver and kidneys of mice

**Figure 3:**
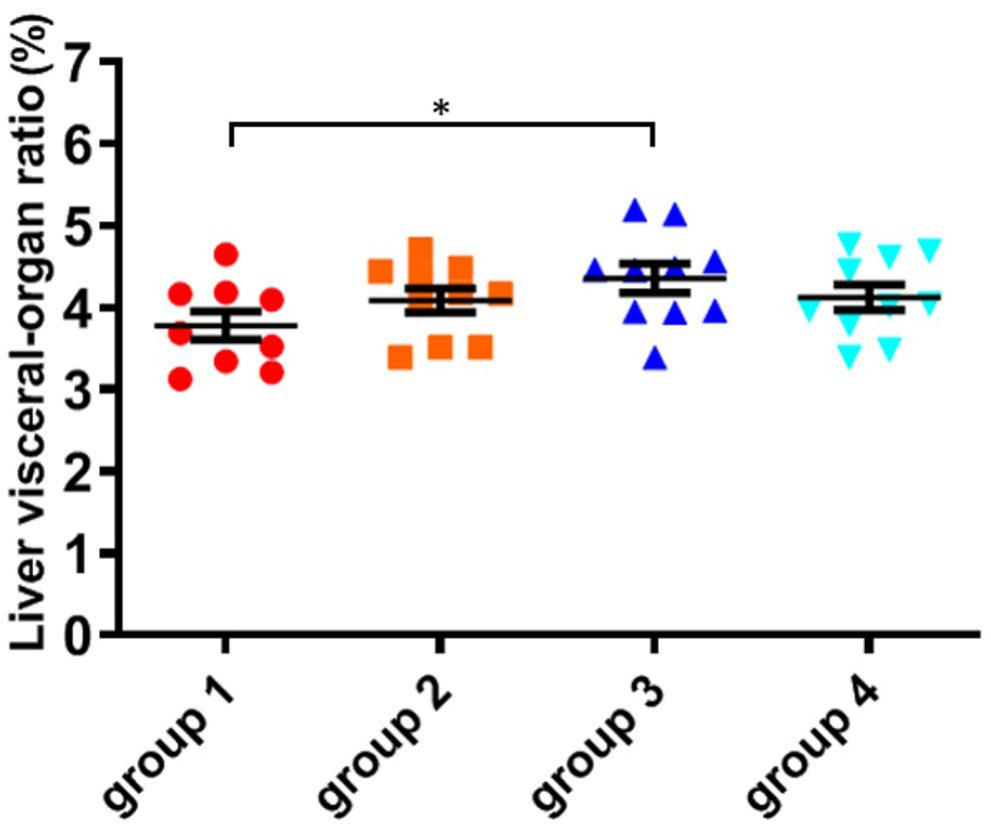
The organ variation coefficient of liver in the four groups, recorded at day 28, when mice were sacrificed. The data is expressed as mean ± standard deviation. *p < 0.05 is significant, analyzed by one-way ANOVA test.

As shown in Figure 3; Table 5, the ratio of liver organ weight:body weight was obtained for four groups of mice that were fed for 28 days. It can be seen that the liver ratio of mice in the third group is significantly higher than that in the control group. Due to the cytotoxicity and immunotoxicity of DON, a high liver ratio of mice may result from swelling, indicating that toxins can damage the liver organs of mice. However, after ozone treatment, the toxin content of the fourth group decreased and its toxicity weakened, with no significant changes in its impact on the liver of mice.

**Table 5:** Relative organ weights of mice at the end of the experiment.

Group	Liver (%)	Kidneys (%)	Spleen (%)
group 1	1.98 ± 0.17^c^	1.63 ± 0.09^a^	0.21 ± 0.06^a^
group 2	2.03 ± 0.09^c^	1.55 ± 0.17^ab^	0.19 ± 0.10^ab^
group 3	2.58 ± 0.23^a^	1.17 ± 0.12^c^	0.13 ± 0.08^c^
group 4	2.25 ± 0.13^b^	1.42 ± 0.14^b^	0.17 ± 0.12^b^

Groups with differing letters are significantly different (adjusted *p* < 0.05).

**Figure 4:**
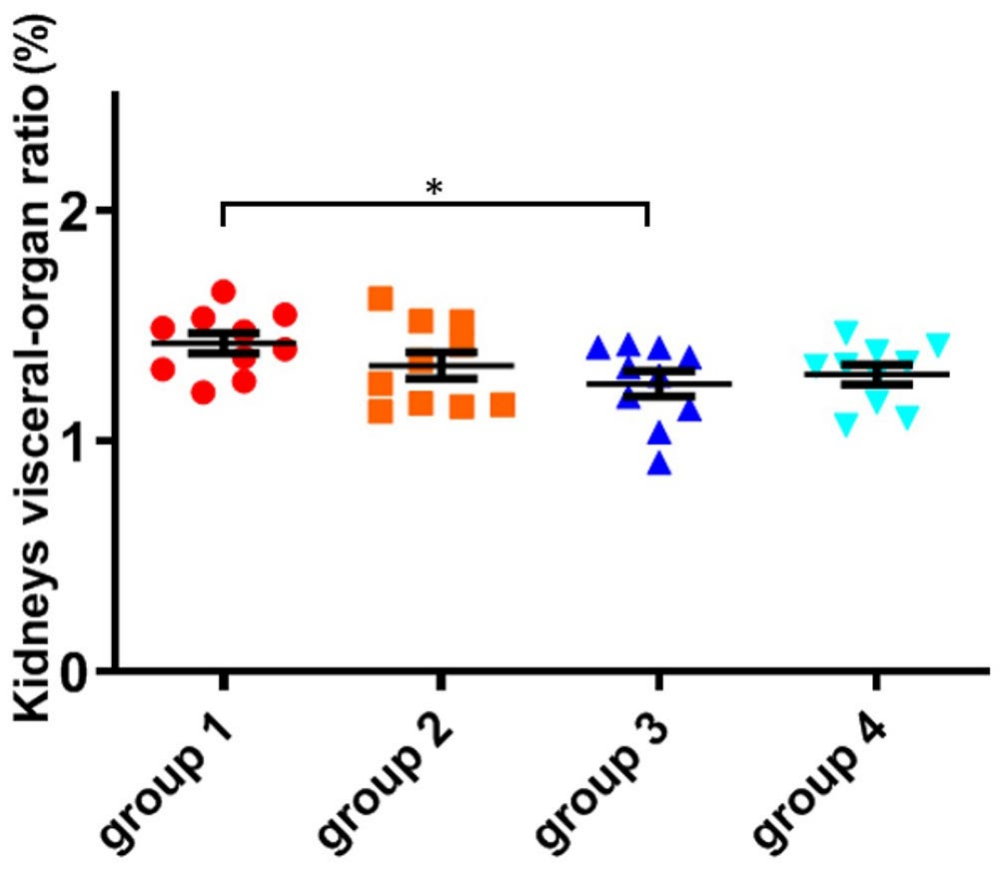
The organ variation coefficient of kidney in the four groups, recorded at day 28, when mice were sacrificed. The data is expressed as mean ± standard deviation. *p < 0.05 is significant, analyzed by one-way ANOVA test.

As shown in Figure 4; Table 5, there was no significant difference in the visceral-organ ratio of the mice kidney between groups 1 (control) and groups 2 (ozone treatment of niblets). However, compared with group 1, the visceral-organ ratio of toxin group 3 (DON-contaminated niblets) was significantly increased, indicating significant changes in the kidney of group 3. The potential cause of kidney swelling in mice could be attributed to the prolonged exposure to corn contaminated with DON over a period of 28 days. The content of DON toxin in the contaminated feed exceeded the national standard limit, ultimately leading to kidney damage in the mice. The results of group 4 (ozone-degrading DON) showed no significant changes in kidney damage compared to group 3. This suggests a decrease in toxin levels after ozone treatment, with no significant negative effects on the kidneys of mice.

From the above results, it can be seen that mice consuming DON-contaminated feed have a certain degree of damage to their livers and kidneys. Ozone treatment of contaminated feed could reduce the damage of DON to mouse livers and kidneys.

### 3.4. Biochemical analysis of serum in mice

According to the blood biochemical indicators of mice in Table 6, there was no significant difference in the levels of total protein, albumin, and globulin between groups 1 and 2. However, group 3 showed a significant decrease in total protein and albumin compared to group 1 (the control, *p* < 0.05), and an increase in globulin values. Albumin is produced in the liver, and its decrease indicates liver damage, which is a direct indicator of the degree of liver and kidney damage. High globulin levels may reflect hepatitis, liver disorders and autoimmune diseases. The corresponding results of group 3 indicates that DON toxin has damaging effects on the livers and kidneys of mice. The results of group 4 showed no significant differences in the indicators of total protein, albumin, and globulin compared to the control group. Under the same dose of toxin, the damage of the toxin decreased after ozone treatment, and there was no significant damage to the mice organs.

**Table 6:** The blood routine examination of experimental groups.

Group	Total protein (g/L)	Albumin (g/L)	Globulin (g/L)	AST (U/L)	ALT (U/L)	BUN (mmol/L)
group 1	57.43 ± 3.12^ab^	42.61 ± 2.31^a^	17.62 ± 1.25^a^	86.29 ± 1.0^d^	32.48 ± 2.19^c^	6.72 ± 0.32^b^
group 2	58.23 ± 2.89^a^	40.07 ± 1.96^ab^	17.35 ± 1.49^a^	85.43 ± 1.01^d^	31.32 ± 2.42^c^	6.64 ± 0.18^b^
group 3	54.61 ± 3.64^bc^	34.25 ± 2.43^d^	20.35 ± 1.21^ab^	96.57 ± 2.0^a^	43.25 ± 2.46^a^	9.06 ± 0.18^a^
group 4	55.57 ± 3.31^c^	38.54 ± 1.33^c^	18.50 ± 1.35^a^	89.32 ± 2.02^c^	35.04 ± 2.53^b^	8.38 ± 0.56^ab^

Groups with differing letters are significantly different (adjusted *p* < 0.05).

Compared with group 1 and group 3, Table 6 showed an abnormal increase in ALT, AST, and BUN reflecting liver and kidney function indicators (*p* < 0.05). Both ALT and AST are present in the liver and can reach the bloodstream through circulation. Therefore, after 28 days of feeding, mice consuming feed containing DON toxin showed significant liver damage. BUN serves as an indicator that reflects the main function of the kidney. The high level of BUN reflects that the kidneys of mice have been damaged, accompanied by some kidney inflammation and other renal dysfunction. The group 4 showed no significant change in detection values compared to group 1. The reduced total protein and albumin levels in serum were similar to the reports related to mice exposed to DON (the same concentration in this study) by gavage (Yang et al., 2021). The group 4 effectively alleviates this inflammatory, immune and hematological changes induced by DON. This finding in mice indicated that the toxicity of DON-contaminated corn after ozone degradation was much lower than that of corn directly contaminated by DON.

### 3.5. Changes in liver, kidney, and thymus tissues of mice

Contaminated food of DON could lead to chronic poisoning in animals due to its cytotoxicity and immunotoxicity after long-term consumption (Ji et al., 2017). It could cause varying degrees of damage to the internal organs of mice, resulting in a series of pathological changes. Therefore, in order to assess the safety of food after ozone degradation, medical pathology tests could be conducted to observe whether DON has an effect on the organs of mice by observing tissue and cell damage through tissue sectioning and cell staining.

**Figure 5:**
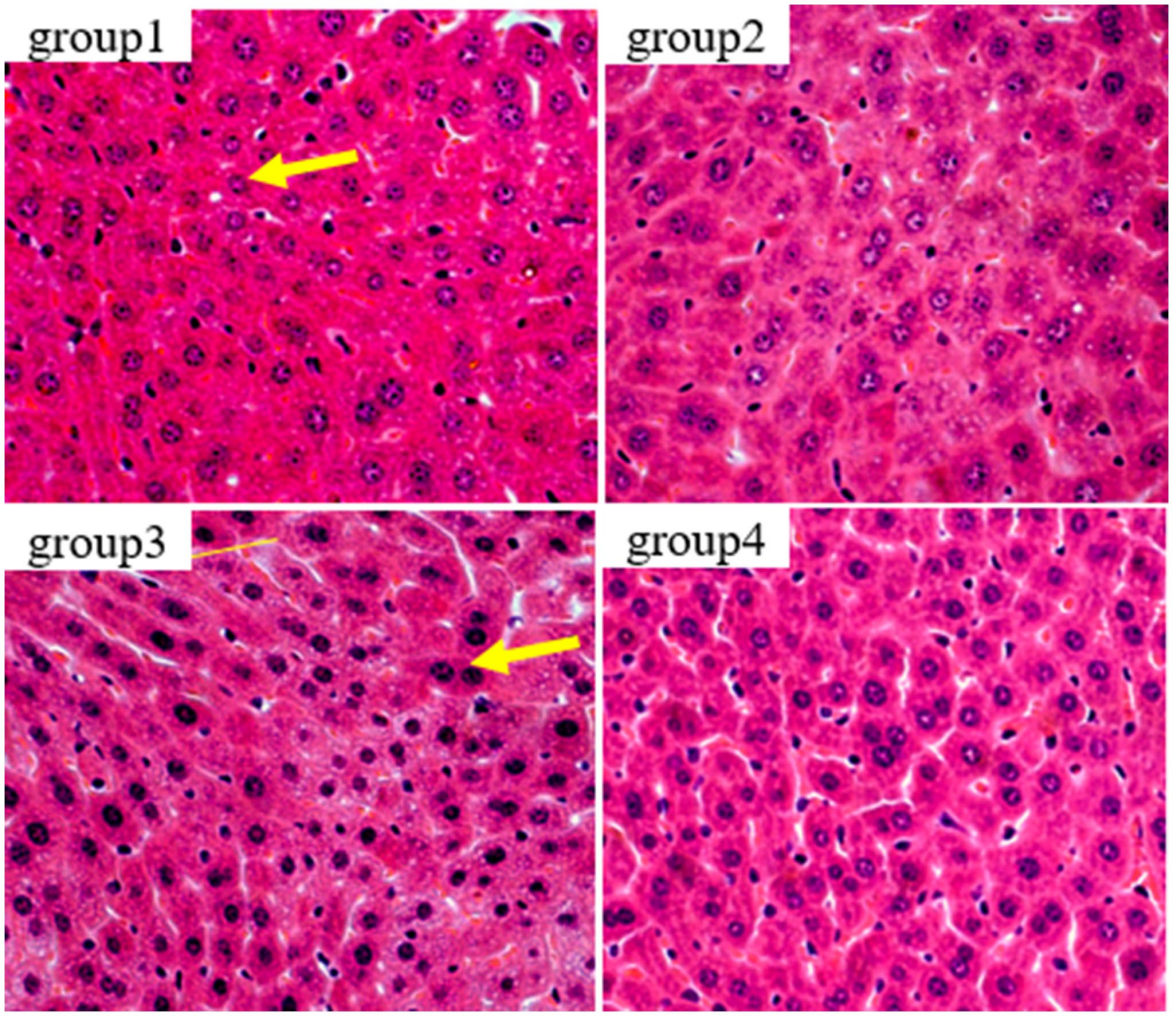
Histopathological examination of control and mycotoxin-treated liver tissues by hematoxylin-eosin staining (magnification: 200×).

In this study, HE staining was used to observe the liver, kidney, and thymus organs of mice. Figure 5 show the results of HE staining sections of mouse liver tissue. Oval cells and lobular structures are clearly visible in the liver tissue of mice in group 1 and 2. The cytoplasm of the liver cells is of normal color, with obvious nuclear and nucleolar structures. The results of group 4 were similar to those of the previous two groups, and there were no significant pathological changes in the mouse liver. From the results of group 3, we found that the liver cells showed significant nuclear enlargement, irregular proliferation of oval cells, alongside disappearance and apoptosis of the nucleus and nucleoli. Also, destruction of the liver lobule structure accompanied by symptoms of epithelial cell necrosis and inflammatory were observed. This indicates that the livers of group 3 of mice were damaged after being treated with DON. This finding further confirms that the DON has detrimental effects to mice.

**Figure 6:**
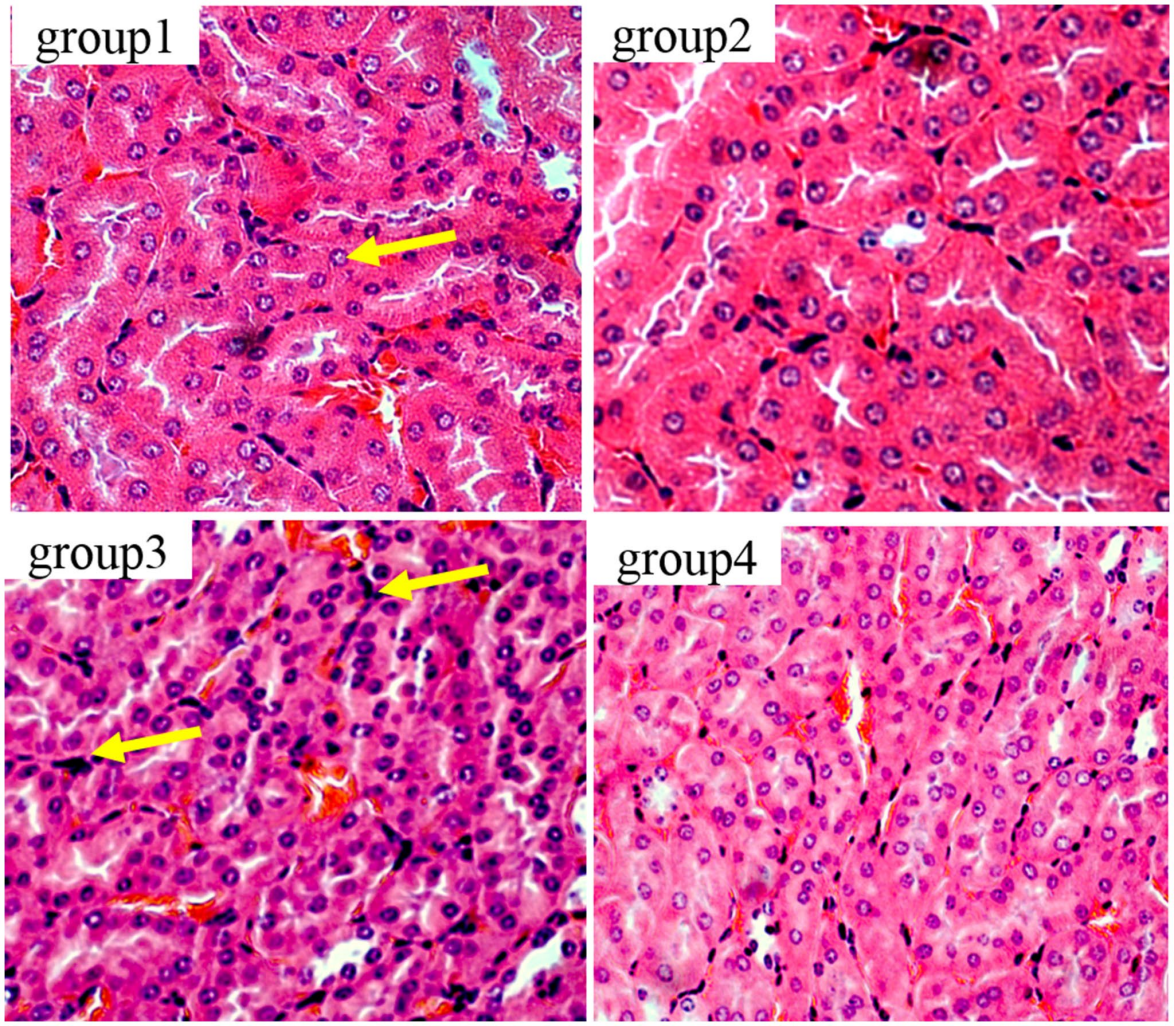
Histopathological examination of control and mycotoxin-treated kidney tissues by hematoxylin-eosin staining (magnification: 200×).

Figure 6 show the results of kidney tissue sections in mice. In the renal structures of groups 1 and 2, the cortical and medullary structures were clear and the shape of the glomerulus was normal. There were no signs of interstitial congestion, and there were no pathological changes such as fibrous tissue proliferation and inflammatory cell infiltration. In group 3, damage to the glomerulus, shrinkage and degeneration of the glomerulus, congestion of the renal interstitium, and tubular necrosis accompanied by interstitial inflammation were detected. The results of group 4 were similar to those of group 1, showing no significant pathological changes, no fibrous tissue hyperplasia, and no interstitial inflammation.

**Figure 7:**
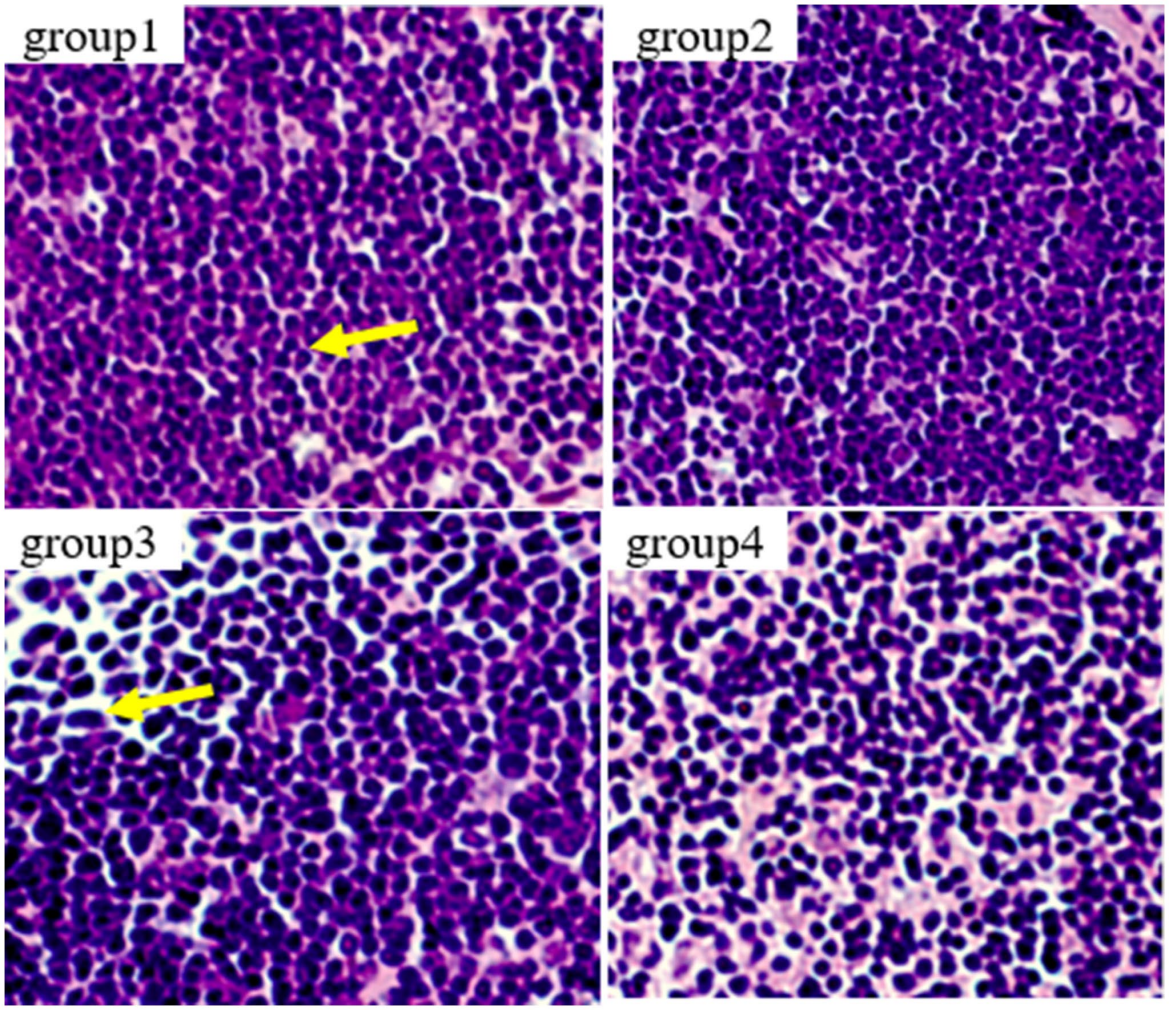
Histopathological examination of control and mycotoxin-treated thymus tissues by hematoxylin-eosin staining (magnification: 200×).

Figure 7 show the results of thymic tissue sectioning in mice. In the thymic structure of groups 1 and 2, the cell structure presents a normal circular or elliptical shape, with clear cortical and medullary structures, normal lymphocytes, and no obvious apoptotic cells. In group 3, thymocytes showed swelling, irregular nuclei, accompanied by polygonal shapes, while lymphocytes increased and the number of macrophages showed mild proliferation.

Altogether, changes in blood physiological and biochemical indicators, and tissue pathological characteristics caused by feeding corn with DON before and after ozone degradation in Kunming mice model were examined, and we demonstrated that the toxicity of DON decreases following this degradation.

## 4. Discussion

In order to investigate the *in vivo* toxicity and assess the toxicity of ozone degradation on DON-contaminated corn, we conducted animal experiments using mice as a simulation. We further analyzed the safety of the treatment by establishing an animal experimental evaluation method for contaminated corn before and after the treatment by simulating the toxin pollution process. The evaluation test shall be conducted according to the national standard 28 days oral toxicity test. We inoculated the toxin-producing *F. graminearum* F7875 into corn kernels. Then, we customize the contaminated corn before and after ozone degradation to prepare a formula that simulates the daily feeding experiment of mice.

In this paper, *F. graminearum* was inoculated onto corn particles to simulate the toxin production test for *F. graminearum* infection. It is more reasonable to develop feeding methods for bio-safety assessment using a mouse model. *F. graminearum* F7875 was inoculated onto corn niblets for solid culture. Following this infection, the previously hard granular corn transformed into fragmented powder. Additionally, the original golden appearance of the corn changed to a brown or reddish color.

Compared to the control group, the Kunming mice in group 3 exhibited a significant decrease in weight after feeding DON-contaminated niblets. However, the DON suffering ozone degradation (group 4) had no significant effect on the weight of the mice. Compared to group 1, there were no significant changes in blood biochemical indicators in groups 2 and 4. However, the indicators in group 3 were abnormal compared to these control groups. This indicates that the toxin has a significant impact on mice health, while this impact is not significant on mice after DON degradation (*p* > 0.05). The mice in group 3 showed significant liver damage after consuming feed containing DON toxin. The high concentration of BUN in group 3 reflects that the kidneys of mice have been damaged, possibly due to kidney inflammation and renal dysfunction. The levels of total protein, albumin, and globulin in groups 2 and 4 were not significantly different from group 1. However, the results of group 3 showed a significant decrease in total protein and globulin compared to group 1 (*p* < 0.05), while globulin values increased. The results of group 3 showed that DON toxin had damaging effects on the livers and kidneys of mice, in accordance with a finding that DON exposure induced immune response in mice (Zong et al., 2023).

## 5. Conclusion

In addition to causing direct acute toxicity, DON toxins also exhibit cellular and immune toxicity. Eating food contaminated with DON can easily cause serious damage to human and animal health, particularly affecting organs such as the livers, kidneys, and intestines. This study simulates the process of *F. graminearum* infecting corn and animal feeding experiments. It mainly follows the 28 days oral toxicity test (archived in national standard GB 15193.22-2014) to study DON toxins, with a little modification. Furthermore, we developed a promising model for in-depth analysis of the effects of DON toxins on animal immunity, metabolism, and health, and measured the *in vivo* toxicity for safety assessment.

## Data availability statement

The original contributions presented in the study are included in the article/supplementary material, further inquiries can be directed to the corresponding author.

## Ethics statement

The animal studies were approved by the ethical principles of the European Community Code. The studies were conducted in accordance with the local legislation and institutional requirements. Written informed consent was obtained from the owners for the participation of their animals in this study.

## Author contributions

CS: Conceptualization, Data curation, Funding acquisition, Project administration, Writing – original draft, Writing – review & editing. FY: Methodology, Writing – original draft. JX: Methodology, Data curation, Writing – original draft. WZ: Investigation, Writing – original draft. JL: Visualization, Writing – review & editing. XG: Data curation, Funding acquisition, Supervision, Writing – review & editing.

## References

[ref1] Al-Jaal B. A. Jaganjac M. Barcaru A. Horvatovich P. Latiff A. (2019). Aflatoxin, fumonisin, ochratoxin, zearalenone and deoxynivalenol biomarkers in human biological fluids: A systematic literature review, 2001–2018. Food Chem. Toxicol. 129, 211–228. doi: 10.1016/j.fct.2019.04.047, PMID: 31034935

[ref2] Awad W. A. Ruhnau D. Hess C. Doupovec B. Schatzmayr D. Hess M. (2019). Feeding of deoxynivalenol increases the intestinal paracellular permeability of broiler chickens. Arch. Toxicol. 93, 2057–2064. doi: 10.1007/s00204-019-02460-3, PMID: 31030221

[ref3] Bai Y. Ma K. Li J. Li J. Bi C. Shan A. (2021). Deoxynivalenol exposure induces liver damage in mice: inflammation and immune responses, oxidative stress, and protective effects of Lactobacillus rhamnosus GG. Food Chem. Toxicol. 156:112514. doi: 10.1016/j.fct.2021.112514 34400200

[ref4] Chen W. Li C. Zhang B. Zhou Z. Shen Y. Liao X.. (2018). Advances in biodetoxification of ochratoxin AA review of the past five decades. Front. Microbiol. 9:1386. doi: 10.3389/fmicb.2018.01386, PMID: 29997599 PMC6028724

[ref5] Deng H. Chen W. Zhang B. Zhang Y. Han L. Zhang Q.. (2023). Excessive ER-phagy contributes to ochratoxin A-induced apoptosis. Food Chem. Toxicol. 176:113793. doi: 10.1016/j.fct.2023.113793, PMID: 37080527

[ref6] Deng Y. You L. Wang X. Wu W. Kuca K. Wu Q.. (2023). Deoxynivalenol: emerging toxic mechanisms and control strategies, current and future perspectives. J. Agric. Food Chem. 71, 10901–10915. doi: 10.1021/acs.jafc.3c02020, PMID: 37437258

[ref7] Edwards S. G. (2009). Fusarium mycotoxin content of UK organic and conventional wheat. Food Addit. Contam. 26, 496–506. doi: 10.1080/02652030802530679 19680924

[ref8] Graziani F. Pujol A. Nicoletti C. Pinton P. Armand L. Di Pasquale E.. (2015). The food-associated ribotoxin deoxynivalenol modulates inducible NO synthase in human intestinal cell model. Toxicol. Sci. 145, 372–382. doi: 10.1093/toxsci/kfv058, PMID: 25766886

[ref9] Hou S. Ma J. Cheng Y. Wang H. Sun J. Yan Y. (2023). The toxicity mechanisms of DON to humans and animals and potential biological treatment strategies. Crit. Rev. Food Sci. Nutr. 63, 790–812. doi: 10.1080/10408398.2021.1954598, PMID: 34520302

[ref10] Ji J. Zhu P. Cui F. Pi F. Zhang Y. Li Y.. (2017). The antagonistic effect of mycotoxins deoxynivalenol and zearalenone on metabolic profiling in serum and liver of mice. Toxins 9:28. doi: 10.3390/toxins9010028, PMID: 28075412 PMC5308260

[ref11] Khaneghah A. M. Martins L. M. von Hertwig A. M. Bertoldo R. Santana A. S. (2018). Deoxynivalenol and its masked forms: characteristics, incidence, control and fate during wheat and wheat based products processing: a review. Trends Food Sci. Technol. 71, 13–24. doi: 10.1016/j.tifs.2017.10.012

[ref12] Pestka J. J. Smolinski A. T. (2005). Deoxynivalenol: toxicology and potential effects on humans. J. Toxicol. Environ. Health B 8, 39–69. doi: 10.1080/10937400590889458 15762554

[ref13] Seyed Toutounchi N. Braber S. Van’t Land B. Thijssen S. Garssen J. Kraneveld A. D.. (2021). Exposure to deoxynivalenol during pregnancy and lactation enhances food allergy and reduces vaccine responsiveness in the offspring in a mouse model. Front. Immunol. 12:797152. doi: 10.3389/fimmu.2021.797152, PMID: 34975906 PMC8718709

[ref14] Sun X. Ji J. Gao Y. Zhang Y. Zhao G. Sun C. (2020). Fate of deoxynivalenol and degradation products degraded by aqueous ozone in contaminated wheat. Food Res. Int. 137:109357. doi: 10.1016/j.foodres.2020.109357, PMID: 33233060

[ref15] Sun C. Zhu P. Ji J. Sun J. Tang L. Pi F.. (2017). Role of aqueous chlorine dioxide in controlling the growth of Fusarium graminearum and its application on contaminated wheat. LWT 84, 555–561. doi: 10.1016/j.lwt.2017.03.032

[ref16] Yang J.-H. Wang J.-H. Guo W.-B. Ling A.-R. Luo A.-Q. Liu D.. (2019). Toxic effects and possible mechanisms of deoxynivalenol exposure on sperm and testicular damage in BALB/c mice. J. Agric. Food Chem. 67, 2289–2295. doi: 10.1021/acs.jafc.8b04783 30707021

[ref1001] Yang Y. Xu Y. Wu S. Qiu T. Blaženović I. Sun J.. (2021). Evaluation of the toxicity and chemical alterations of deoxynivalenol degradation products under ozone treatment. Food Control. 124:107937. doi: 10.1016/j.foodcont.2021.107937

[ref17] Ye Y. Jiang M. Hong X. Fu Y. Chen Y. Wu H.. (2023). Quercetin alleviates Deoxynivalenol-induced intestinal damage by suppressing inflammation and Ferroptosis in mice. J. Agric. Food Chem. 71, 10761–10772. doi: 10.1021/acs.jafc.3c02027, PMID: 37392437

[ref18] Zha A. Liao S. Tan B. Liao P. (2023). Integrated lnc RNA transcriptomics, proteomics, and metabolomics to identify early cellular response variation in deoxynivalenol-treated IPEC-J2 cells. Food Chem. Toxicol. 177:113863. doi: 10.1016/j.fct.2023.113863 37257635

[ref19] Zhao X. Sun P. Liu M. Liu S. Huo L. Ding Z.. (2022). Deoxynivalenol exposure inhibits biosynthesis of milk fat and protein by impairing tight junction in bovine mammary epithelial cells. Ecotoxicol. Environ. Saf. 237:113504. doi: 10.1016/j.ecoenv.2022.113504, PMID: 35447471

[ref20] Zhou Y. Qi S. Meng X. Lin X. Duan N. Zhang Y.. (2021). Deoxynivalenol photocatalytic detoxification products alleviate intestinal barrier damage and gut flora disorder in BLAB/c mice. Food Chem. Toxicol. 156:112510. doi: 10.1016/j.fct.2021.112510, PMID: 34390814

[ref21] Zong Q. Li K. Qu H. Hu P. Xu C. Wang H.. (2023). Sodium butyrate ameliorates Deoxynivalenol-induced oxidative stress and inflammation in the porcine liver via NR4A2-mediated histone acetylation. J. Agric. Food Chem. 71, 10427–10437. doi: 10.1021/acs.jafc.3c02499, PMID: 37384814

